# Dissolution and Interaction of Cellulose Carbamate in NaOH/ZnO Aqueous Solutions

**DOI:** 10.3390/polym13071092

**Published:** 2021-03-30

**Authors:** Yanhui Kang, Fangyu Wang, Zeming Zhang, Jinping Zhou

**Affiliations:** Hubei Engineering Center of Natural Polymers-Based Medical Materials, Key Laboratory of Biomedical Polymers of Ministry of Education, Department of Chemistry, Wuhan University, Wuhan 430072, China; kangyanhui0707@163.com (Y.K.); wangfangyu9527@gmail.com (F.W.); zmzhang0224@163.com (Z.Z.)

**Keywords:** cellulose carbamate, NaOH/ZnO aqueous solution, freezing–thawing method, dissolution interaction

## Abstract

The dissolution and molecular interactions of cellulose carbamate (CC) in NaOH/ZnO aqueous solutions were studied using optical microscopy, differential scanning calorimetry (DSC), ^1^H NMR, dynamic light scattering (DLS), atomic force microscopy (AFM), transmission electron microscopy (TEM), and molecular dynamic simulation. The dissolution of CC in NaOH/ZnO aqueous solutions using the freezing–thawing method was an exothermic process, and the lower temperature was favorable for the dissolution of CC. ZnO dissolved in NaOH aqueous solutions with the formation of Zn(OH)_4_^2−^, and no free Zn^2+^ ions existed in the solvents. NaOH/Na_2_Zn(OH)_4_ system formed strong interactions with the hydroxyl groups of CC to improve its solubility and the stability of CC solution. The results indicate that 7 wt% NaOH/1.6 wt% ZnO aqueous solution was the most appropriate solvent for the dissolution of CC. This work revealed the dissolution interaction of CC-NaOH/ZnO solutions, which is beneficial for the industrialization of the CarbaCell process.

## 1. Introduction

It is well known that the rapidly growing awareness of environmental pollution has shifted researchers’ focus from traditional petroleum-derived synthetic polymers to more environmentally friendly alternatives [1]. As a widely available reproducible organic material, cellulose is a promising substitute to fossil resources because of its renewability, nontoxicity, and environmental friendliness [2]. Furthermore, it has been developed for thousands of years in the production of fiber, paper, film, filters, and textiles [3,4,5]. However, a large number of intra- and intermolecular hydrogen bonds of cellulose hinder dissolution, which is an impediment to its wide utilization [6]. Nevertheless, the derivatization of cellulose can improve its solubility and enable new functions and applications [7]. Cellulose xanthogenate is an ancient viscose technology that has been used for more than 100 years [8,9]. The traditional viscose process consumes significant time and energy, is expensive, and produces harmful by-products such as CS_2_, H_2_S, and heavy metals [10,11]. The development of environmentally friendly systems and processes for the regenerated cellulose industry has been highly valuable. To reduce processing steps and minimize harmful by-products, multiple innovative methods and novel solvents for cellulose have been developed [12,13,14,15,16,17]. However, commercially viable processes are rare.

The CarbaCell process is a promising alternative to the conventional viscose method, and employs cellulose carbamate (CC) as an active intermediate for fiber spinning [18,19]. Cellulose reacts with urea to produce CC, which is soluble in NaOH aqueous solutions [20]. The use of innocuous urea avoids the problem associated with hazardous sulfur-containing compounds. Several means exist to synthesize CC, including esterification reaction in N, N-dimethylacetamide (DMAc) [21], the isocyanate-pyridine procedure [22], the “pad-drycure” method [23], supercritical CO_2_-assisted impregnation [24,25], and electron radiation [26]. In previous work, we reported the fast synthesis of CC in several minutes by microwave heating without using a solvent or catalyst [27,28,29]. More recently, the low content of urea aqueous solution was used to soak cellulose, and CC was synthesized by conventional heating [30,31]. CC can be dissolved in 9 wt% NaOH aqueous solution [28]. However, a rheological study showed that CC/NaOH aqueous solutions were extremely prone to forming gel at room temperature [27]. To improve the solubility of CC and the stability of spinning dope, researchers have conducted extensive research. For example, it has been found that the addition of a small amount of urea to NaOH aqueous solution can increase the solubility of CC and reduce the viscosity of the solution [20]. CC can also be dissolved in precooled 18 wt% NaOH solution under intensive stirring [32]. However, none of these methods have achieved industrial CarbaCell production. Therefore, the dissolution of CC is particularly important for green and sustainable development [33].

Cellulose can be dissolved in NaOH [34], NaOH/urea [17] and NaOH/zinc nitrate hexahydrate aqueous solutions [35] using the freezing–thawing method to obtain transparent solutions. This can be ascribed to the partial cleavage of hydrogen bonds between the hydroxyl groups of cellulose [35]. Similarly, the desired amount of CC can be dissolved into the NaOH/ZnO aqueous system, and a transparent CC solution can also be obtained by the freezing–thawing method [36]. We found that adding a small amount of ZnO to NaOH aqueous solution can significantly improve the solubility of CC and the stability of the spinning dope. The regenerated cellulose filaments, membranes, and nanocomposites have been successfully prepared from the CC solutions [37,38]. However, the interactions of CC-NaOH/ZnO aqueous solutions have not been explored systematically to date. In this work, the dissolution and interaction of CC in NaOH/ZnO aqueous solutions were investigated in detail. In NaOH aqueous systems, ZnO existed in the form of Zn(OH)_4_^2−^ hydrates. NaOH/Na_2_Zn(OH)_4_ hydrates can form stronger interactions with the hydroxyl groups of CC compared with the sole NaOH hydrates, thus promoting the dissolution of CC. These stronger interactions may include H-bonding, ionic, and electrostatic interactions.

## 2. Experimental Section

### 2.1. Materials

Cotton linter pulps with α-cellulose content of more than 95% were provided by Hubei Chemical Fiber Co. Ltd. (Xiangyang, China) as the cellulose raw materials. The *M*_η_ in cadoxen was determined by viscometry to be 7.4 × 10^4^ g/mol. NaOH, urea, and ZnO of analytical grade (Shanghai Chemical Reagent Co. Ltd., Shanghai, China) were used directly. Deionized water was used throughout the whole experiment.

### 2.2. Oven Heating Synthesis of CC

**Scheme 1:**
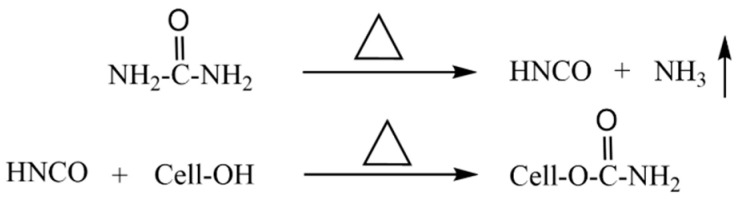
The synthesis process of CC from cellulose and urea.

According to the previous work [38,39], every 100 g of cotton linter pulp was immersed into 2000 g of urea solution with the concentration of 3 wt%. To make the cotton linter pulps fully immersed in the urea solution, the mixtures were stirred every 1 h at 25 °C for 8 h, then filtered, and vacuum dried. Subsequently, the mixtures of cellulose/urea with the urea content of 3.5 wt% were obtained. The cellulose/urea mixtures were heated in an oven at 132.7 °C in a sealed container for 30 min and then heated to 160 °C for 60 min. Finally, the mixtures were washed with deionized water and vacuum-dried at 50 °C. The synthesis of CC from cellulose and urea was as Scheme 1 [18]:

### 2.3. Dissolution of CC

Based on previous work [30], NaOH and ZnO were directly dissolved in distilled water to obtain the solvents. The contents of NaOH and ZnO were varied from 5 to 10 wt% and 0 to 2 wt%, respectively. ZnO is an amphoteric oxide, which exists as Zn(OH)_4_^2−^ in the alkali aqueous solution: ZnO + 2OH^−^ + H_2_O → Zn(OH)_4_^2−^. The specific composition of the solvent is shown in Table S1.

Subsequently, the desired amount of CC was dispersed into the solvent, cooled to −24 °C in the refrigerator to form a solid for 8 h, and then thawed at ambient temperature. The dissolved and insoluble portions in the solutions were separated by centrifugation at 7000 rpm for 10 min to obtain the clear CC solution.

### 2.4. Characterizations

The intrinsic viscosity ([η]) of CC in cadoxen at 25 °C was determined with an Ubbelohde viscometer [40], and the viscosity-average molecular weight (*M*_η_) was determined to be 7.0 × 10^4^ g/mol. The nitrogen content of CC was ascertained to be 0.832%, which was measured using an elemental analyzer (CHN-O-RAPID Hereaus Co., Hanau, Germany). Then, 500 mL NaOH/ZnO aqueous solution was placed in a glass bottle and sealed for 30 days, waiting for the crystal to precipitate out. Then we poured the solvent out and used the spoon to scrape the crystals off the bottle wall. We washed them with distilled water and froze them to dry. X-ray diffraction (XRD) measurement was performed using an XRD diffractometer (D8-Advance, Bruker, Karlsruhe, Germany). The pattern of Cu Kα radiation (λ = 0.15418 nm) at 40 kV and 30 mA was recorded in the 2θ region from 10 to 80° at a scanning speed of 4°/min.

An optical microscope (ZEISS AXIO SCOPE A1POL) was used to observe the dissolution of CC in the solvent as the freezing time extended. The CC and solvent mixtures were taken out of the refrigerator at different freezing times. Subsequently, an appropriate amount of mixture was directly pressed between the two glass slides, then sealed with paraffin, observed, and photographed. The solubility of CC was determined by observing the CC solutions with the polarized mode. After a certain concentration of CC was obtained, the solution was subjected to polarized microscope observation at room temperature. The area observed in the polarized microscope was a 20 mm diameter circular field. When no fibers were observed, CC was considered to be dissolved at that concentration. Each solubility was tested at least 3 times to confirm the accuracy and repeatability.

Differential scanning calorimetry (DSC) measurements were made using a TA Q20 instrument (New Castle, DE, USA). CC was dispersed in NaOH (5–10 wt%)/ZnO (0–1.6 wt%) hydrates and sealed in a stainless pan. The temperature was programmed from 20 to −40 °C and then from −40 to 20 °C at a rate of 1 °C/min under N_2_ atmosphere. ^1^H NMR spectra were measured on a Varian INOVA-600 spectrometer in the proton noise-decoupling mode at 25 °C. The chemical shifts of protons were referenced to the signals of D_2_O and tetramethylsilane (TMS). The CC concentrations for DSC and ^1^H NMR measurements were 5 wt% and 0.3 wt%, respectively.

The hydrodynamic radius (R_h_) distributions of CC in NaOH/ZnO aqueous solutions were characterized by dynamic light scattering (DLS) on a multi-angle light scattering spectrometer (ALV/SP-125, ALV, Langen, Germany) equipped with an ALV-5000/E multi-digital time correlator. The scattering angle was 90°. The solutions were kept for 10 min at each condition before measurement. All of the CC solutions (c = 0.3 g/L) were optically cleaned through 0.45 and 0.22 µm Millipore filters (Whatman, Inc., Clifton, NJ, USA).

Atomic force microscope (AFM) images were measured with an AFM (Cypher ES, Asylum Research, Santa Barbara, CA, USA) in ac mode at 25 °C. Silicon probes with a spring constant of 2 N/m and resonance frequency of 70 kHz (OLTESPA-R3, Bruker, Billerica, MA, USA) were employed. All data from the images were analyzed using AFM accessory software, and the images presented were flattened only when necessary. The CC solution was purified by a 0.22 µm Millipore filter (Whatman, Inc., Clifton, NJ, USA), deposited onto the wafer, and air-dried overnight before imaging. TEM images were obtained using a JEM-2010(HT) transmission electron microscope (JEOL TEM, Tokyo, Japan). Before TEM observation, the CC solution was cast onto a perforated carbon film, which was supported on a copper grid, and air-dried overnight. The concentration of CC for AFM and TEM measurements was 1.0 × 10^−4^ g/mL. To make the image clearer, we use distilled water to gently moisten the surface of the sample to remove excess salt particles.

Molecular dynamic simulation software was used to confirm the interaction between CC and solvents. The calculation was performed using Amber 3 force field as implemented in Hyper Chem 8.0.10. A set of 0.1 Kcal/(Å·mol) and a conjugate gradient method (Polak–Ribiere) were used for geometric optimization. During energy minimization, the molecular structure was simulated, in which small changes in geometry can give the most stable configuration. Modeling helped to explore the effect of the non-covalent interactions, specifically the hydrogen bond in the growing polymer chains. The volume of the periodic analog box was 12 × 10 × 8 Å. In this simulation, 10 repeating units were calculated and Zn^2+^, Na^+^, OH^−^ and H_2_O were introduced into the simulation process. The composition of the force field in the simulation included covalent bonds, angles, and twists, in addition to the interaction of electrostatic and hydrogen bonds [41,42,43].

## 3. Results and Discussion

**Figure 1:**
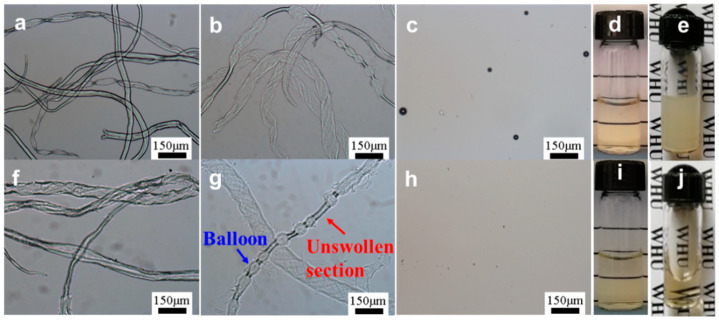
Optical microscopic observation of cellulose carbamate (CC) dissolving process in (**a**–**e**) 7 wt% NaOH and (**f**–**j**) 7 wt% NaOH/1.6 wt% ZnO aqueous solutions at −24 °C for different duration times: (**a**,**f**) 0 min; (**b**,**g**) 5 min; (**c**,**h**) 10 min. (**d**,**i**) the new CC solutions and (**e**,**j**) CC solutions placed at room temperature for 12 h.

The optical microscopic images of CC dissolved in 7 wt% NaOH and 7 wt% NaOH/1.6 wt% ZnO solutions with various freezing times are shown in Figure 1. After soaking for a short time at room temperature (freezing 0 min), the morphology of CC, both in 7 wt% NaOH and 7 wt% NaOH/1.6 wt% ZnO aqueous solutions, was not obviously different. CC was lightly swelled in the solvent, and the diameters of fibers were in the range of 15–70 μm (Figure 1a,f). The degree of swelling and dispersion augmented as the freezing time increased from 0 to 5 min. It has been reported that cellulose could expand in strong alkaline media [44,45]. CC began to expand on the fibers’ isolated points, forming balloons (Figure 1b,g). The area of the balloons inflated, resulting in the appearance of bead-like structures. The diameter of the fibers expanded until they broke. After the balloons exploded, CC solution coexisted with the undissolved CC in the mixed system. When the freezing time reached 10 min, both CC in 7 wt% NaOH and 7 wt% NaOH/1.6 wt% ZnO systems had analogous clear microscope images and transparent solutions were obtained (Figure 1c,h), indicating the complete dissolution of CC. Although CC can be dissolved in a 7 wt% NaOH system (Figure 1d), the obtained CC/7 wt% NaOH solutions easily changed into gel due to the unstable interaction of CC-NaOH (Figure 1e), and the sol-gel transition was a thermo irreversible process [27]. However, the CC/7 wt% NaOH/1.6 wt% ZnO solutions can be stable for a long period (Figure 1i,j).

By adding a small amount of ZnO to the NaOH aqueous solutions, the solubility of CC was also significantly improved. Figure S1 shows the solubility of CC increased with an increasing mass fraction of NaOH (5–10 wt%) and ZnO (0–1.6 wt%), whereas the solubility of CC decreased in 7 wt% NaOH/2 wt% ZnO solution. The 7 wt% NaOH/2 wt% ZnO solution was unstable due to the excess of ZnO. As shown in Figure S2, the crystals of orthorhombic Zn(OH)_2_ (JCPDS 38-0385) will precipitate out from the solutions over the storage time at room temperature. The maximum solubility (9 wt%) of CC was obtained in the 7 wt% NaOH/1.6 wt% ZnO system. NaOH aqueous solution can not only dissolve CC, but also provide conversion of ZnO to Zn(OH)_4_^2−^, thus improving the solubility of CC and maintaining the stability of CC solution. NaOH/ZnO solutions possessed stronger dissolving capacity than the pure NaOH solution, suggesting that Zn(OH)_4_^2−^ performed the auxiliary role in the dissolution of CC at the lower temperature.

**Figure 2:**
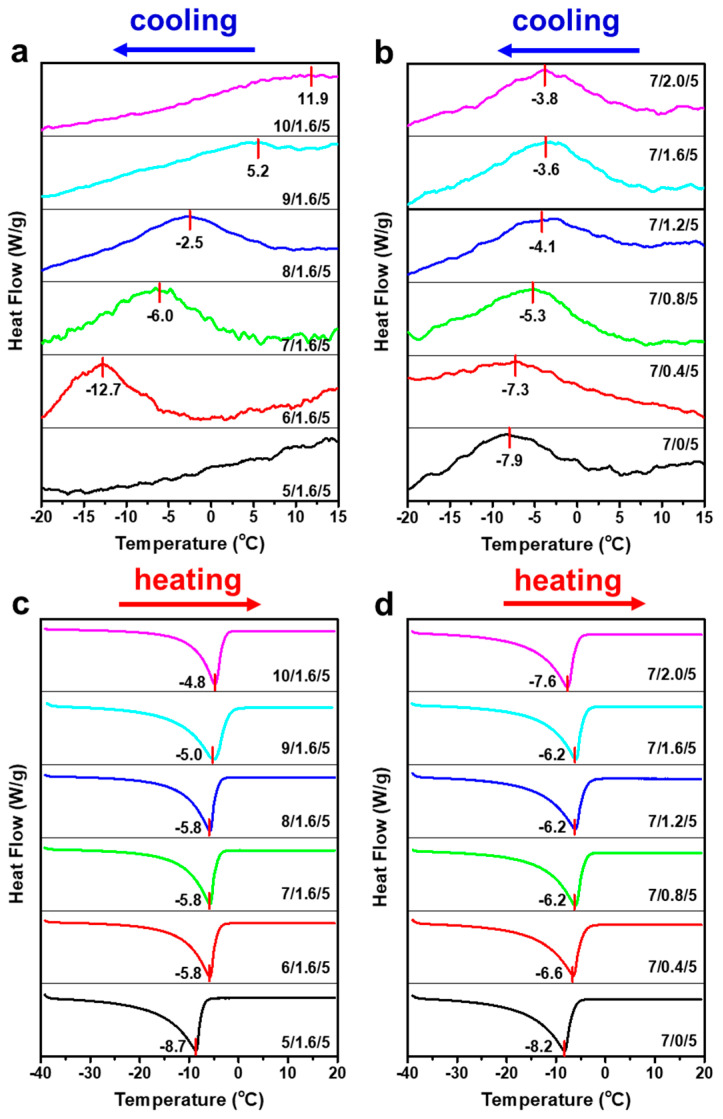
Magnified DSC (**a**,**b**) cooling and (**c**,**d**) heating thermograms of 5 wt% CC in NaOH/ZnO aqueous solutions with various NaOH and ZnO contents.

Dissolution of cellulose has been proven to be a process involved with the thermal effect [46,47,48]. Figure 2 shows the magnified DSC curves for the cooling (from 15 to −20 °C) and heating (from −40 to 20 °C) processes of the CC mixed with NaOH/ZnO systems. It is worth noting that the subtle exothermic peaks for the dissolution of CC were observed at the temperature range of 15 to −20 °C. When the ZnO content was 1.6 wt%, the dissolution enthalpy peaks of CC gradually shifted from −12.7 to 11.9 °C as the NaOH content increased from 6 to 10 wt% (Figure 2a), suggesting that the interaction between CC and solvents gradually enhanced with an increase in the NaOH content [47]. The dissolution enthalpy peaks of CC in 5 wt % NaOH/1.6 wt% ZnO aqueous solution could not be observed because it was lower than −20 °C. The dissolution of CC in NaOH/ZnO aqueous solution indicated a representative exothermic enthalpy-driven process. In the heating process, the melting enthalpy peaks of CC/NaOH/ZnO frozen mass shifted slightly from −8.7 to −4.8 °C as the NaOH content increased from 5 to 10 wt% (Figure 2c). Analogously, when the NaOH content was 7 wt%, the dissolution enthalpy peaks of CC shifted slightly from −7.9 to −3.6 °C as the ZnO content increased from 0 to 1.6 wt% (Figure 2b). As a result that the stability of 7 wt% NaOH/2.0 wt% ZnO solvent was lowered, the dissolution enthalpy peak of CC shifted to −3.8 °C. Moreover, the melting enthalpy peaks of CC/NaOH/ZnO frozen mass gradually shifted from −8.2 to −6.2 °C as the ZnO content increased from 0 to 1.6 wt%, and then shifted to −7.6 °C as the ZnO content further increased to 2.0 wt% (Figure 2d).

**Figure 3:**
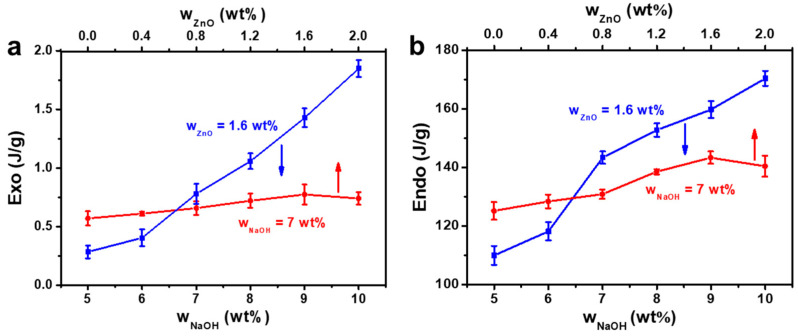
(**a**) The dissolution enthalpies of CC and (**b**) The melting enthalpies of CC/NaOH/Na_2_Zn(OH)_4_/H_2_O frozen mass as a function of NaOH and ZnO contents.

The corresponding dissolution enthalpies of CC and melting enthalpies of CC/NaOH/Na_2_Zn(OH)_4_ systems are shown in Figure 3. When the ZnO content was 1.6 wt% and the NaOH content increased from 5 to 10 wt%, the dissolution enthalpies of CC increased from 0.28 to 1.85 J/g, and the melting enthalpies of CC/NaOH/Na_2_Zn(OH)_4_/H_2_O frozen mass increased from 118.2 to 170.4 J/g. When the NaOH content was 7 wt% and the ZnO content increased from 0 to 1.6 wt%, the dissolution enthalpies of CC gradually increased from 0.58 to 0.78 J/g. Similarly, the melting enthalpies of the frozen mass gradually increased from 128.5 to 143.4 J/g. There was a conspicuous transition in the dissolution and melting enthalpy curves in the light of ZnO content, located at the mass fraction of 7 wt% NaOH/1.6 wt% ZnO. This mass proportion was considered to be the optimum ratio for the dissolution of CC. After further increasing the ZnO content to 2.0 wt%, the dissolution enthalpy of CC and melting enthalpy of CC/NaOH/Na_2_Zn(OH)_4_/H_2_O frozen mass declined slightly to 0.74 and 140.4 J/g, respectively. Therefore, excessive ZnO was not conducive to the dissolution of CC. The melting enthalpies were related the amount of melting structures. As shown in Table S1, the quantity of NaOH and Na_2_Zn(OH)_4_ hydrates in the solvent increased with increasing NaOH (5–10 wt%) and ZnO (0–1.6 wt%) content. Therefore, the quantity of CC/NaOH/Na_2_Zn(OH)_4_ hydrates also increased when CC was added into the solvent.

**Figure 4:**
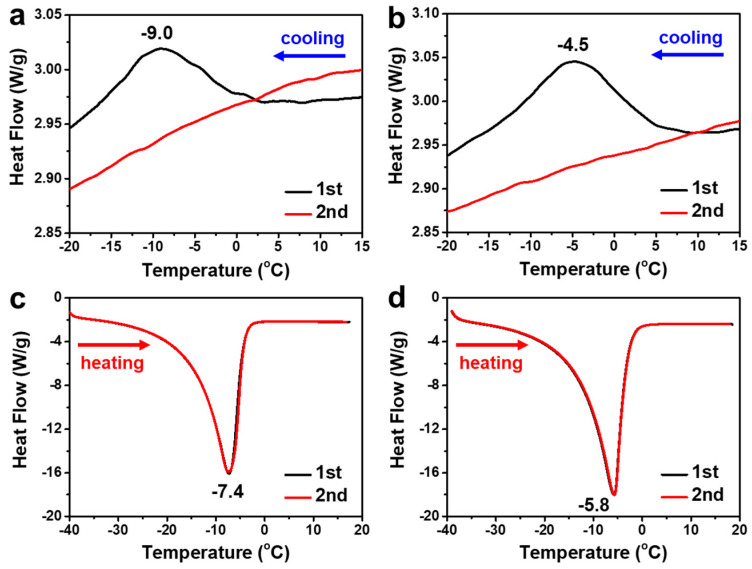
Magnified DSC profiles of cooling and heating thermograms of 5 wt% CC in (**a**,**c**) 7 wt% NaOH and (**b**,**d**) 7 wt% NaOH/1.6 wt% ZnO aqueous solutions.

The magnified DSC profiles of the cooling and heating processes during two cycles of CC (5 wt%) in 7 wt% NaOH and 7 wt% NaOH/1.6 wt% ZnO systems are displayed in Figure 4. It is worth noting that the dissolution enthalpy peaks of CC only appeared in the first cooling cycle, both in NaOH and NaOH/ZnO aqueous solutions (−9.0 and −4.5 °C, respectively). No dissolution enthalpy peak occurred in the second cooling process at the temperature range from 15 to −20 °C, indicating that the complete dissolution of CC was achieved by merely cooling once (Figure 4a,b) [49,50]. In the heating profiles of CC/NaOH and CC/NaOH/ZnO frozen mass, sharp melting enthalpy peaks appeared at −7.4 and −5.8 °C, respectively (Figure 4c,d). Figure S3 shows the melting enthalpies of CC/NaOH and the CC/NaOH/Na_2_Zn(OH)_4_ frozen mass. Whether the first or second cycle, the melting enthalpy of the CC/NaOH/Na_2_Zn(OH)_4_ frozen mass is larger than that of the CC/NaOH frozen mass. When the CC concentration is varied from 1 to 5wt% (Figure S4a,b), the melting enthalpy peaks gradually shifted from −6.9 to −6.2 °C (CC/NaOH frozen mass) and −5.7 to −4.1 °C (CC/NaOH/ZnO frozen mass). Furthermore, the corresponding melting enthalpies (Figure S4c) also increase from 117.1 to 135.6 J/g and 141.0 to 144.8 J/g, respectively.

**Figure 5:**
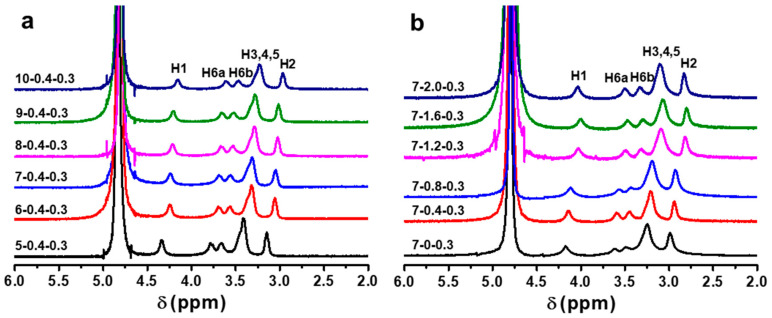
^1^H NMR spectra of CC (c = 0.3 wt%) in NaOH/ZnO/D_2_O with different (**a**) NaOH and (**b**) ZnO contents.

The chemical shifts of NMR spectra are sensitive to the formation and breakage of hydrogen bonding [51]. The proton shift strongly depends on the concentration of electrolytes when they are added into water [52]. Hence, ^1^H NMR was used to study the structure and interaction of CC in the solvent systems (Figure 5). Due to the rapid proton exchange, the chemical shift at 4.78 ppm belonged to the protons of D_2_O in the coaxial capillaries as an external reference. The signals distributed in the range of 2.5–4.5 ppm corresponded to the protons of glucose rings of CC [53]. The chemical shifts of H1 (4.3 ppm), H6a (3.8 ppm), H6b (3.6 ppm), H3,4,5 (3.4 ppm), and H2 (3.1 ppm) for CC in 5 wt% NaOH/0.4 wt% ZnO/D_2_O were larger than those of H1 (4.1 ppm), H6a (3.6 ppm), H6b (3.4 ppm), H3,4,5 (3.2 ppm), and H2 (3.0 ppm) for CC in 10 wt% NaOH/0.4 wt% ZnO wt%/D_2_O. Clearly, the signals of the protons shifted upfield with an increase in the NaOH content (Figure 5a) [54,55]. Their upfield shift is likely a consequence of a higher extent of hydrogen bonding and deprotonation involving −OH groups exerting thus a shielding effect on the C-H protons. The OH^−^ ion acted as strong hydrogen bonding acceptors and formed hydrogen bonding with −OH groups in alkali solutions. The higher the concentration of NaOH, the stronger the hydrogen bonding interaction between OH^−^ ion and −OH group, resulting in the stronger electro-shielding effect of CC [56]. Therefore, the proton resonances were shifted upfield with an increase in the NaOH concentration. Figure 5b shows the ^1^H NMR spectra of CC solutions with different ZnO contents. The chemical shifts of the protons in 7 wt% NaOH/D_2_O solutions were H1 (4.2 ppm), H6a (3.6 ppm), H6b (3.5 ppm), H3,4,5 (3.2 ppm), and H2 (3.0 ppm). The signal of the protons moved to the higher field as the ZnO content increased from 0 to 1.6 wt%. After adding ZnO to NaOH(aq), Zn(OH)_4_^2^^−^ acted as stronger proton acceptors than OH^−^, the deprotonation effects of −OH groups increased in NaOH/ZnO(aq), and the electro-shielding effect of the protons also increased. As a result, the signals of protons moved upfield upon the addition of ZnO. Moreover, when the ZnO contents were in the range of 1.2–2.0 wt%, the resonances of protons hardly changed, which were H1 (4.0 ppm), H6a (3.5 ppm), H6b (3.3 ppm), H3,4,5 (3.0 ppm), and H2 (2.8 ppm), respectively. Therefore, the ZnO content did not have further influence on the chemical shifts, and thus did not have further influence on the interaction between CC and solvent system. Compared with Figure 5a,b, we can conclude that the hydrogen-bonding and deprotonation effect between CC and the NaOH/ZnO system are stronger than those between CC and the NaOH system.

**Figure 6:**
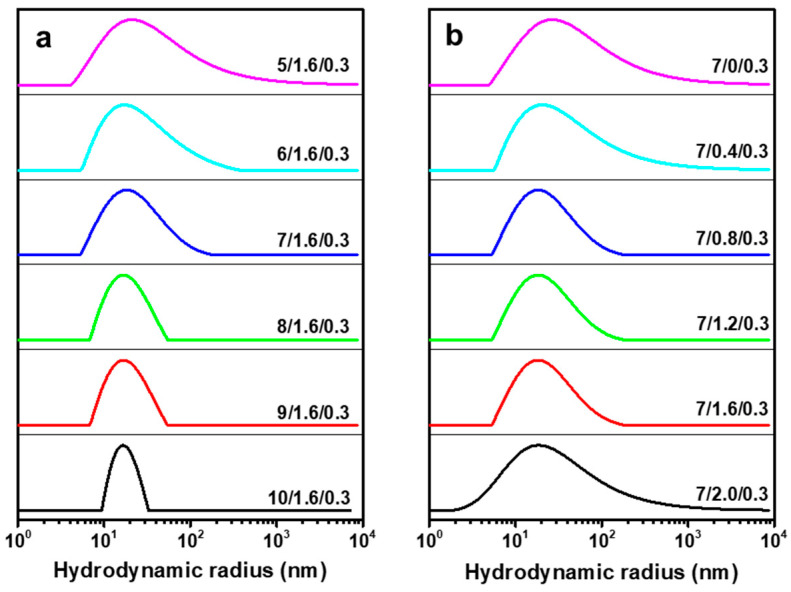
Hydrodynamic radius (R_h_) distributions of CC (c = 0.3 g/L) in NaOH/ZnO aqueous solutions with various contents of (**a**) NaOH and (**b**) ZnO at 25 °C.

The dissolved state of CC in NaOH/ZnO aqueous solutions was also verified by DLS, which was evidenced by its R_h_ distributions. Figure 6 shows the R_h_ distributions of CC in various solvent systems. Only one symmetrical peak existed in the CC solutions. As the NaOH content increased from 5 to 10 wt%, the R_h_ distributions of CC gradually narrowed, suggesting that the higher concentration of OH^−^ was beneficial to the dissolution of CC (Figure 6a). As displayed in Figure 6b, the R_h_ distributions of CC gradually narrowed as the ZnO content increased from 0 to 1.6 wt%, suggesting that CC was uniformly dissolved in the solvent system. However, when the ZnO content reached 2.0 wt%, the peak was wide, indicating that the excessive ZnO caused uneven dissolution of CC. According to our experiment, Zn(OH)_2_ crystals gradually precipitated out of the CC solution when the solvent was 7 wt% NaOH/2.0 wt% ZnO hydrates. An appropriate amount of ZnO added to the 7 wt% NaOH solution could dissolve a greater quantity of CC. However, excessive ZnO added to the 7 wt% NaOH solution would worsen the dissolution effect. Figure S5 shows the R_h_ values of CC in NaOH/ZnO aqueous solutions with various NaOH and ZnO contents. When the ZnO content was 1.6 wt%, the R_h_ values of CC decreased from 37.7 ± 2.2 to 18.0 ± 1.4 nm as the NaOH content increased from 5 to 10 wt%. When the NaOH content was fixed at 7 wt%, the R_h_ values of CC decreased from 43.7 ± 2.8 to 21.4 ± 1.6 nm as the ZnO content increased from 0 to 1.6 wt%. When the ZnO content attained 2.0 wt%, the R_h_ of CC chains increased to 29.0 ± 2.1 nm due to the instability of the solution. R_h_ reflects the solvatization of CC [57]. The stronger the solvation effect, the more thoroughly the CC was dissolved. This is because the more solvent molecules that CC was exposed to, the better the dissolution of CC at low temperature. Based on the above results, considering that excess NaOH was not conducive to large-scale production in the plant, it was reasonable to believe that 7 wt% NaOH/1.6 wt% ZnO solution was the most appropriate solvent for CC.

**Figure 7:**
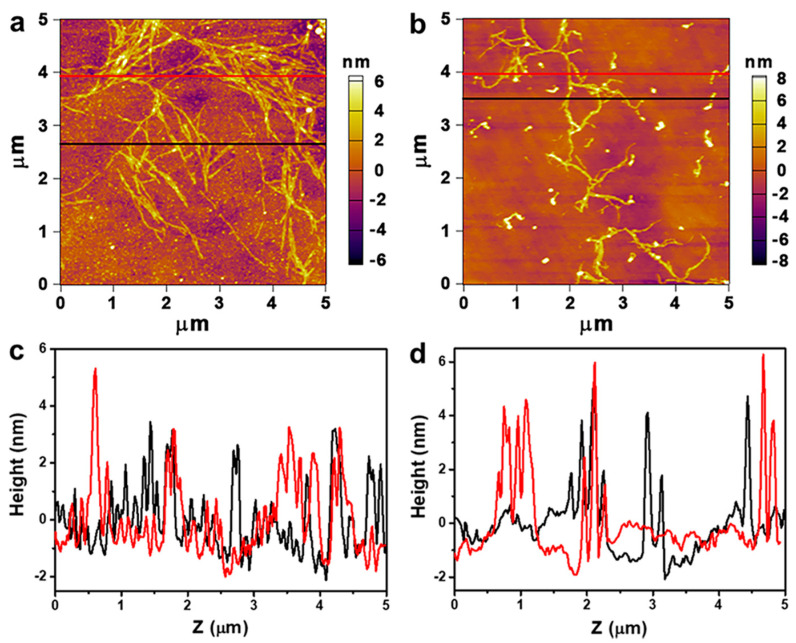
Atomic force microscopy (AFM) images of CC in (**a**) 7 wt% NaOH and (**b**) 7 wt% NaOH/1.6 wt% ZnO aqueous solutions (c = 1.0 × 10^−4^ g/mL). (**c**,**d**) the height distributions of CC as indicated by the red and black lines in (**a**,**b**).

**Figure 8:**
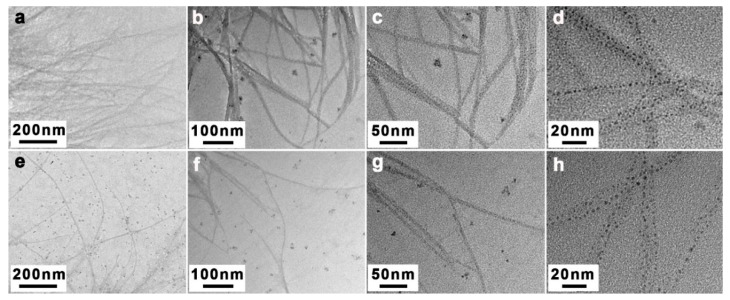
TEM images of CC in (**a**–**d**) 7 wt% NaOH and (**e**–**h**) 7 wt% NaOH/1.6 wt% ZnO aqueous solutions (c = 1.0 × 10^−4^ g/mL).

Under strictly controlled and identical drying conditions, the CC solutions lose water and the CC shrink during the drying process, resulting in the aggregation of nanofibers [51,58]. To indirectly explore the dissolving effect of CC in NaOH and NaOH/ZnO aqueous solutions, the morphology of CC dried from the dilute solution was measured by AFM. As shown in Figure 7a, the aggregation state of CC was obvious. In contrast, Figure 7b shows the better dissolved state of CC. The heights indicated by red and black lines in the AFM images were estimated to be 1.0 to 6.0 nm (Figure 7c,d), which could be defined as the diameter of the CC aggregates [59,60]. The results indicate that the NaOH/ZnO aqueous solution has greater dissolving effect for CC than the NaOH aqueous solution. To provide additional indirect evidence on the dissolution of CC in NaOH and NaOH/ZnO aqueous solutions, TEM was used to observe their morphology. TEM images of the CC dilute solution dried at room temperature are shown in Figure 8. As expected, the images displayed better dissolved state in Figure 8e–h than in Figure 8a–d. It was suggested that the 7 wt% NaOH/1.6 wt% ZnO system had better dissolution ability for CC than the 7 wt% NaOH system. The results further confirm that Zn(OH)_4_^2−^ hydrates played the auxiliary role in the dissolution of CC.

**Figure 9:**
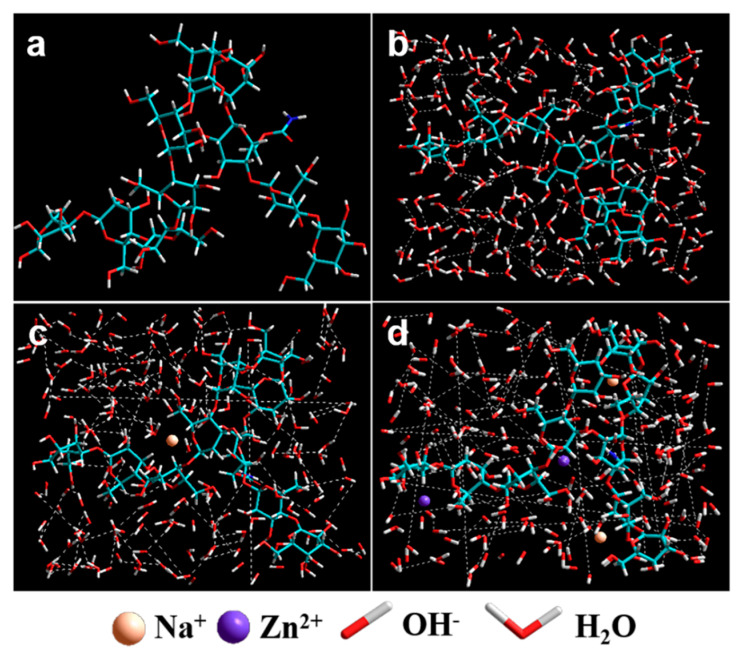
The molecular simulation of (**a**) CC chains, CC in (**b**) H_2_O, (**c**) NaOH/H_2_O, and (**d**) NaOH/ZnO/H_2_O.

**Figure 10:**
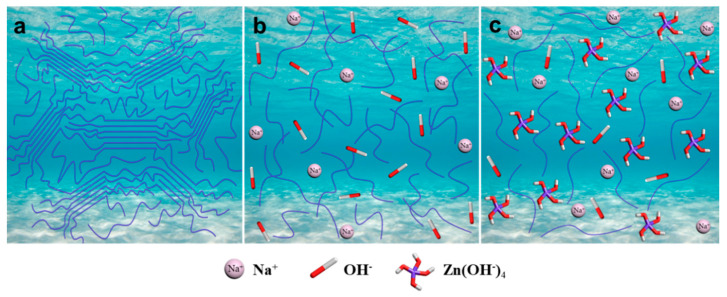
Schematic illustration for the dissolution mechanism of CC in (**a**) H_2_O, (**b**) NaOH/H_2_O, and (**c**) NaOH/ZnO/H_2_O.

To further confirm the above discussion on the interactions of CC-NaOH/ZnO solutions, molecular dynamic simulation via modeling software was used [61]; the results are shown in Figure 9. As can be seen, hydroxyl groups of CC and NaOH in the solvent formed new hydrogen bonds. Compared with H_2_O (Figure 9a) and NaOH solution (Figure 9b), CC can form a more intensive hydrogen bond network in NaOH/ZnO solution (Figure 9c), leading to intermolecular hydrogen bond cleavage of CC. The dissolution mechanism of CC in NaOH/ZnO aqueous solution is illustrated in Figure 10. In H_2_O system (Figure 10a), the crystal structure of the CC chains did not change. The R_h_ values of CC in 7 wt% NaOH and 7 wt% NaOH/1.6 wt% ZnO aqueous solutions were determined to be 43.7 ± 2.8 and 21.4 ± 1.6 nm by DLS, respectively. The radii of Na^+^, Zn^2+^ and OH^−^ were 0.095, 0.074 and 0.176 nm, respectively [62]. Under the effect of small molecules of solvents, the hydrogen bonds of CC were broken at low temperature to obtain a clear solution. In the NaOH/H_2_O system (Figure 10b), NaOH maintained the breaking of the hydrogen bonding of CC. Na^+^ combined with water molecules to form hydrated ions. When CC was dissolved in NaOH/H_2_O solutions, CC chains were further combined with Na^+^ hydrate through electrostatic interaction. The overall effects in the system result in an exothermic dissolution process for CC. However, the interactions of CC-NaOH were metastable in NaOH aqueous solution, which was easily entangled and gelled at room temperature. Adding ZnO to the NaOH aqueous solution, ZnO transferred into Zn(OH)_4_^2−^ hydrates, and CC-NaOH/ZnO interactions could be formed in the solution (Figure 10c). Compared with the NaOH hydrates, NaOH/ZnO hydrates can form stronger interactions with the hydroxyl groups of CC, resulting in enhanced dissolution ability and the stability of the spinning dope.

## 4. Conclusions

CC can be dissolved in NaOH/ZnO aqueous systems via a freezing–thawing method to form transparent solutions. This was found to be a typical enthalpy-driven process, and low temperature was favorable for the dissolution of CC. With the increase of NaOH and ZnO contents, the amount of hydrates increased; the interaction of CC-NaOH and CC-NaOH/ZnO systems also increased. Thus, the melting enthalpies of the frozen mass increased. ZnO existed as Zn(OH)_4_^2−^ hydrates in the NaOH aqueous solution. Compared with the sole NaOH system, the NaOH/Na_2_Zn(OH)_4_ systems significantly improved the solubility of CC and the stability of CC solution, due to the strong interactions between the Zn(OH)_4_^2−^ hydrates and the hydroxyl groups of CC. Therefore, 7 wt% NaOH/1.6 wt% ZnO aqueous solution was proven to be the most appropriate solvent for CC, and this work provides a theoretical basis for the application of the CarbaCell process.

## Data Availability

The data presented in this study are available on request from the corresponding author.

## Supplementary Materials

The following are available online at https://www.mdpi.com/article/10.3390/polym13071092/s1, Table S1: Composition and parameters of NaOH/ZnO aqueous solutions; Figure S1: Solubility of CC in NaOH/ZnO aqueous solutions with various NaOH and ZnO contents, Figure S2: XRD pattern of the crystals precipitated out from 7 wt% NaOH/2 wt% ZnO and CC/7 wt% NaOH/2 wt% ZnO aqueous solution over the storage time at room temperature, Figure S3: The melting enthalpies of 5 wt% CC in 7 wt% NaOH and 7 wt% NaOH/1.6 wt% ZnO aqueous solutions during the 1st and 2nd cooling/heating processes (20 °C~−40 °C~20 °C),Figure S4: Magnified DSC heating thermograms of CC (1, 3 and 5 wt%) in (a) 7 wt% NaOH and (b) 7 wt% NaOH/1.6 wt% ZnO aqueous solutions; (c) the corresponding melting enthalpies of the CC solutions, Figure S5: The hydrodynamic radius (Rh) of CC in NaOH/ZnO aqueous solutions with various NaOH and ZnO contents were calculated from the CONTIN analysis.
