# Supplementary material for: Dissolution and Interaction of Cellulose Carbamate in NaOH/ZnO Aqueous Solutions
Source: Polymers (Basel). 2021 Mar 30;13(7):1092. doi: 10.3390/polym13071092 (PMC8037852; doi:10.3390/polym13071092)
Supplement: Supplementary file 1 [file polymers-13-01092-s001.pdf]

polymers

Table S1. Composition and parameters of NaOH/ZnO aqueous solutions.

Solvent			Hydrates in 100 g of solvent		Concentration of Hydrate	
w_{NaOH} (wt%)	w_{ZnO} (wt%)	ρ (g/mL)	NaOH (mol)	$Na_2Zn(OH)_4$ (mol)	NaOH (mol/L)	$Na_2Zn(OH)_4$ (mol/L)
5	1.6	1.0658	0.0857	0.01967	0.9130	0.2096
6	1.6	1.0726	0.1107	0.01967	1.1874	0.2110
7	1.6	1.0875	0.1357	0.01967	1.4757	0.2139
8	1.6	1.0947	0.1607	0.01967	1.7592	0.2153
9	1.6	1.1052	0.1857	0.01967	2.0524	0.2174
10	1.6	1.1098	0.2107	0.01967	2.3383	0.2183
7	0	1.0697	0.1750	0	1.8720	0
7	0.4	1.0812	0.1652	0.00491	1.7861	0.0531
7	0.8	1.0832	0.1553	0.00983	1.6822	0.1065
7	1.2	1.0857	0.1455	0.01474	1.5797	0.1600
7	2.0	1.0914	0.1259	0.02457	1.3741	0.2682

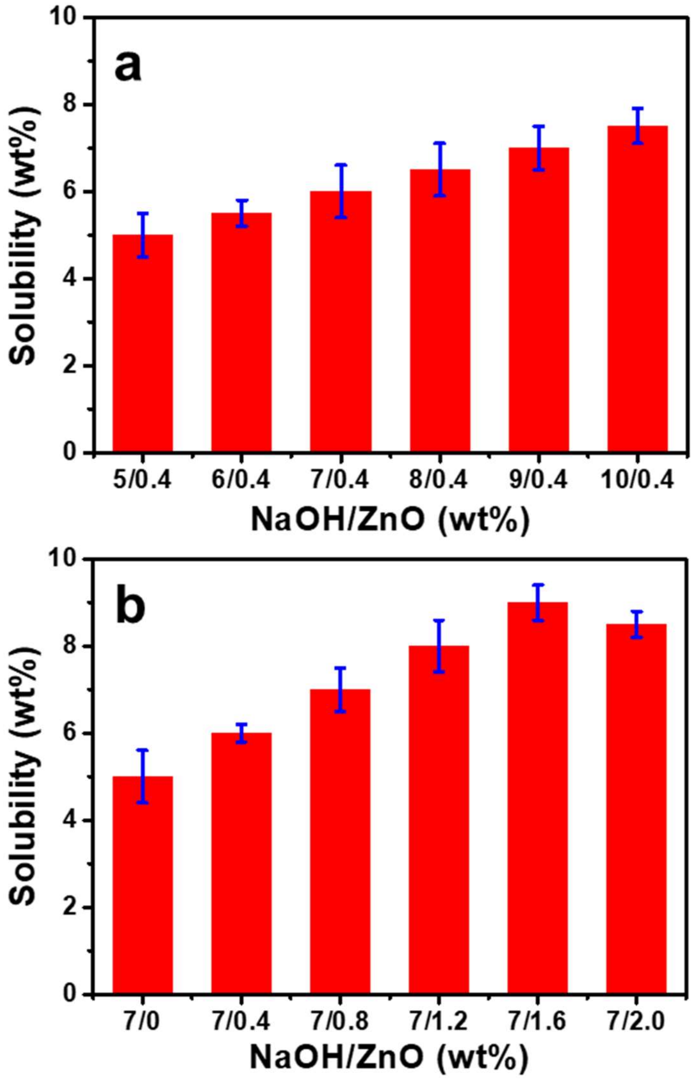

Figure S1. Solubility of CC in NaOH/ZnO aqueous solutions with various NaOH and ZnO contents.

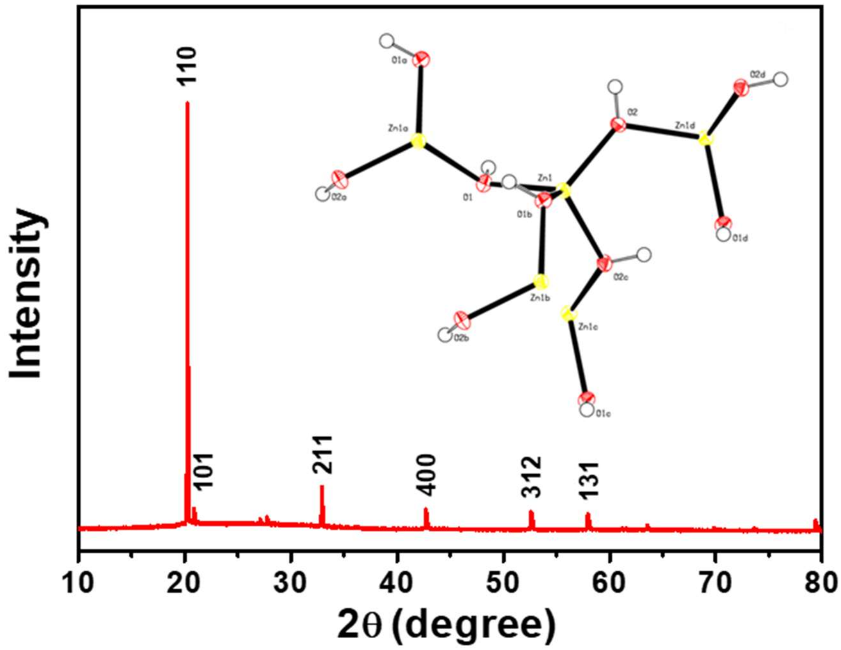

Figure S2. XRD pattern of the crystals precipitated out from 7 wt% NaOH/2 wt% ZnO and CC/7 wt% NaOH/2 wt% ZnO aqueous solution over the storage time at room temperature.

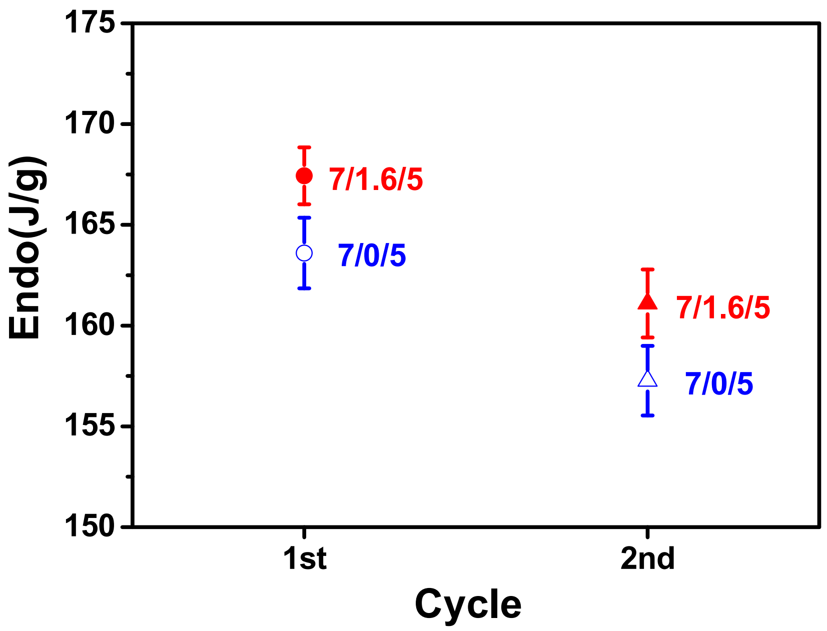

Figure S3. The melting enthalpies of 5 wt% CC in 7 wt% NaOH and 7 wt% NaOH/1.6 wt% ZnO aqueous solutions during the 1st and 2nd cooling/heating processes (20 °C ~ -40 °C ~ 20 °C).

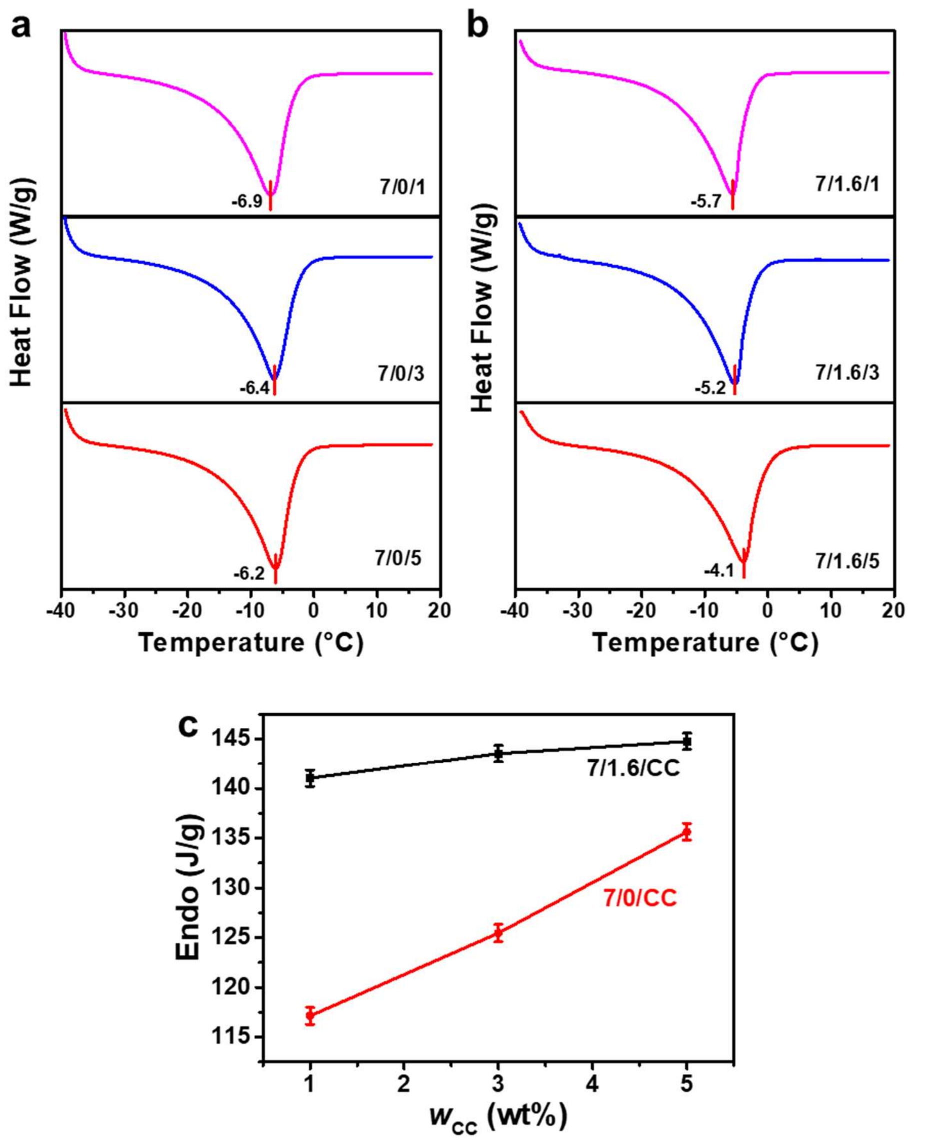

Figure S4. Magnified DSC heating thermograms of CC (1, 3 and 5 wt%) in (a) 7 wt% NaOH and (b) 7 wt% NaOH/1.6 wt% ZnO aqueous solutions; (c) the corresponding melting enthalpies of the CC solutions.

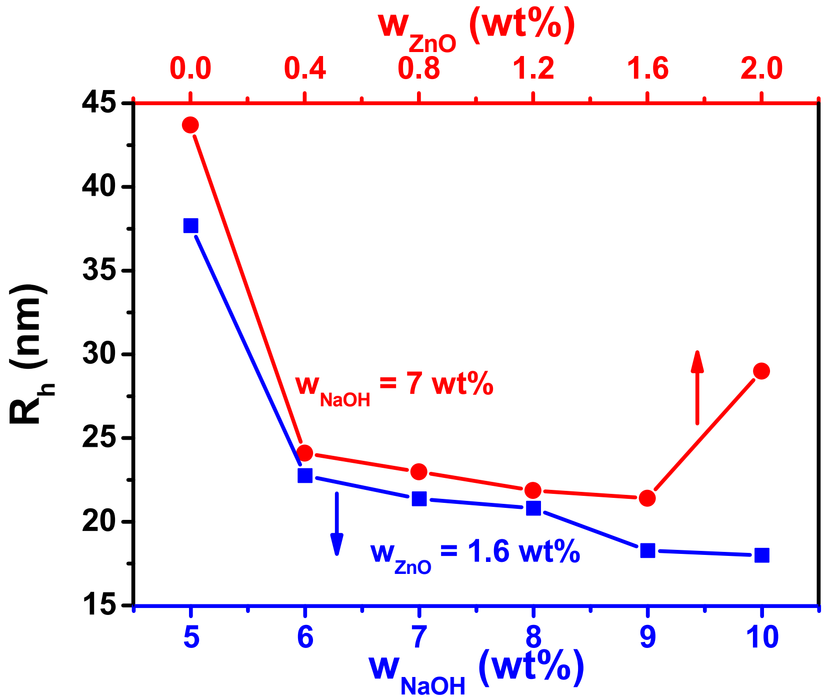

Figure S5. The hydrodynamic radius (R_h) of CC in NaOH/ZnO aqueous solutions with various NaOH and ZnO contents were calculated from the CONTIN analysis.
